# MicroRNA-195-3p inhibits cyclin dependent kinase 1 to induce radiosensitivity in nasopharyngeal carcinoma

**DOI:** 10.1080/21655979.2021.1979356

**Published:** 2021-09-29

**Authors:** Fuchuan Xie, Wei Xiao, Yunming Tian, Yuhong Lan, Chi Zhang, Li Bai

**Affiliations:** Department of Radiation Oncology, Huizhou Municipal Central Hospital, Guangdong, China

**Keywords:** Nasopharyngeal carcinoma, radiotherapy, miR-195-3p, cyclin-dependent kinase 1

## Abstract

MicroRNAs (miRNAs) are revealed to participate in the progression of multiple malignancies, including nasopharyngeal carcinoma (NPC). This work is intended to decipher the function of microRNA-195-3p (miR-195-3p) in regulating the radiosensitivity of NPC cells and its mechanism. MiR-195-3p and cyclin-dependent kinase 1 (CDK1) expressions were detected in NPC tissues and cells using qRT-PCR and Western blot, respectively. Moreover, radiation-resistant cell lines were induced by continuous irradiation with different doses. Furthermore, the CCK-8 experiment, colony formation assay and flow cytometry were utilized to examine the growth, apoptosis and cell cycle of radioresistant cells. Bioinformatics prediction and dual-luciferase reporter gene assay were applied to prove the targeting relationship between miR-195-3p and CDK1 mRNA 3ʹUTR. The data showed that miR-195-3p was remarkably down-modulated in NPC tissues and was associated with increased tumor grade, lymph node metastasis and clinical stage of the patients. MiR-195-3p expression was significantly down-modulated in radiation-resistant NPC tissues and NPC cell lines relative to radiation-sensitive NPC tissues and human nasopharyngeal epithelial cells, while CDK1 expression was notably up-modulated. MiR-195-3p overexpression inhibited the growth of NPC cells, decreased radioresistance, promoted apoptosis, and impeded the cell cycle progression. CDK1 was a target gene of miR-195-3p, and CDK1 overexpression counteracted the effects of miR-195-3p on NPC cell growth, apoptosis, cell cycle progression and radiosensitivity. In summary, miR-195-3p improves the radiosensitivity of NPC cells by targeting and regulating CDK1.

## 1. Introduction

Nasopharyngeal carcinoma (NPC) is a prevailing malignancy in Southern China, with the high morbidity and mortality [1,2]. NPC is considered as a malignancy sensitive to radiation therapy [3]. However, after long-term radiation exposure, tumor cells express certain genes and proteins abnormally and become less radiosensitive, which eventually leads to tumor recurrence or disease progression [4]. Therefore, clarifying the mechanism of radioresistance of NPC cells, and improving the effect of NPC radiotherapy is of great significance.

MicroRNA (miRNA) is short endogenous non-coding RNAs containing 21–25 nucleotides in length with tissue specificity [5]. To date, about 11,000 miRNAs have been identified in human, and they are crucial regulators in biological processes [6]. MiRNA is implicated in cancer cells’ malignant biological characteristics, such as radioresistance [7]. The function of miR-195-3p in tumors is well studied. For instance, miR-195-3p is lowly expressed in oral tumor, and miR-195-3p overexpression inhibits angiogenesis and migration of cancer cells [8]. In hepatocellular carcinoma, miR-195-3p under-expression is closely linked to unfavorable prognosis of the patients, and miR-195-3p overexpression significantly inhibits tumor cell migration and invasion [9]. Importantly, the role of some miRNAs in the radiosensitivity of NPC cells has been reported, such as miR-483-5p, miR-206 and miR-24 [10–12]. However, the role of miR-195-3p in NPC radiosensitivity is still obscure.

In this work, bioinformatics implies that cyclin-dependent kinase 1 (CDK1) may be a target gene of miR-195-3p. We hypothesized that miR-195-3p may regulate the radiosensitivity of NPC cells via modulating CDK1. This study was executed to verify the scientific hypothesis mentioned above.

## 2. Materials and methods

### 2.1 Ethics committee and clinical tissue specimen collection

Cancer tissue specimens from 99 NPC patients (50 radiotherapy-sensitive patients and 49 radiotherapy-resistant patients) admitted to Huizhou Municipal Central Hospital from April 2017 to April 2020, and another 46 cases of normal tissues specimens were collected during biopsy and rapidly in liquid nitrogen. For the NPC patients, case inclusion criteria: (1) diagnosis of NPC with pathological examination after the biopsy; (2) no treatment such as radiotherapy and chemotherapy before the biopsy; (3) without serious inflammation or necrosis in the lesion. Case exclusion criteria: (1) patients received any anti-cancer treatments before the biopsy; (2) patients who did not agree to donor the samples; (3) patients with other malignancy or inflammatory diseases. The procedure was authorized by the Ethics Committee of Huizhou Municipal Central Hospital.

### 2.2 Regents, kits and materials

RPMI-1640 medium, penicillin, and streptomycin was obtained from Gibco, Carlsbad, CA, USA. Fetal bovine serum (FBS) was obtained from HyClone, GE Healthcare Life Sciences, Little Chalfont, UK. Overexpression vectors and miRNA mimics were obtained from GenePharma Co., Ltd., Shanghai, China. Lipofectamine^TM^ 2000 and TRIzol kit were obtained from Invitrogen, Carlsbad, CA, USA. miScript Reverse Transcription Kit and miScript SYBR Green PCR system were obtained from QIAGEN, GmbH, Hilden, Germany. PrimeScript RT kit was obtained from Takara, Dalian, China. SYBR Green Master Mix kit was obtained from Takara, Otsu, Japan. Cell counting kit-8 (CCK-8) was obtained from Beyotime, Shanghai, China. Annexin V-FITC/propidium iodide (PI) double-labeled staining kit was obtained from Yeasen Biotech Co., Ltd., Shanghai, China. Dual-Luciferase Reporter Assay System was obtained from Promega, Madison, WI, USA. RIPA lysis buffer was obtained from Pierce, Rockford, IL, USA. BCA Protein Quantification Assay Kit was obtained from Pierce, Rockford, IL, USA. The antibodies were obtained from Abcam, Cambridge, MA, USA. PVDF membranes were obtained from Millipore, Billerica, MA, USA. Electrochemiluminescence kit was obtained from Biosharp, Hefei, China.

### 2.3 Cell culture and transfection

NPC cell lines (5–8 F, 6–10B, CNE1, CNE2, C666-1) and normal nasopharyngeal epithelial cell line NP69 were available from the Cancer Institute of Southern Medical University (Guangzhou, China). The cells were cultured in RPMI-1640 medium containing 10% FBS, 100 U/mL penicillin, 100 μg/mL streptomycin at 37°C in 5% CO_2_. The medium was changed every 3–4 days, when the confluence of the cells reached to about 60%.

To induce the radioresistance of NPC cells, Varian 2300EX linear accelerator (Varian, Palo Alto, CA, USA) was used to perform the radiation. Wild type 5–8 F and 6–10B NPC cells in logarithmic growth phase were irradiated with increasing doses of radiotherapy (1, 2, 4, 6, 8 Gy). The cells were treated with each dose at least 3 times until the cells were resistant to the radiation, and then the next dose was used. The radioresistant NPC cells were named by 5–8 F-R and 6–10B-R.

To perform transfection, the cells were trypsinized with 0.25% trypsin (Roche, Basel, Switzerland). After the trypsinization, the density of the cells was modulated to 1 × 10^5^ cells/ml with serum-free RPMI-1640 medium. Afterward, the cells were planted into 6-well plates (2 ml/well) and cultured for 24 h. MiR-195-3p mimics, negative control mimics (NC mimics), CDK1 overexpression plasmid, and control plasmid were dissolved in serum-free medium, and mixed with Lipofectamine^TM^ 2000. Thereafter, the mixture was supplemented into the wells, and cells were transfected for 4 h. Then the transfection system was removed, and the cell culture was continued for 24 h with fresh complete medium. 24 h later, the transfection efficiency was determined by qRT-PCR.

### 2.4 qRT-PCR

Total RNA was isolated using TRIzol reagent, and reverse transcribed to cDNA using miScript Reverse Transcription Kit and PrimeScript RT kit. miScript SYBR Green PCR system and SYBR Green Master Mix kit were utilized to perform PCR on a Rotorgene 6,000 real-time PCR machine (Corbett Research, Sydney, Australia). U6 or GAPDH were regarded as the internal references. MiR-195-3p and CDK1 mRNAs’ relative expression was analyzed using the 2^−ΔΔCt^ technique. The primer sequences are as follows: miR-195-3p forward primer:5ʹ-CCAATATTGGCTGTGCTGCTCC-3ʹ, reverse primer:Universal primer (miScript SYBR Green PCR kit); CDK1 forward primer:5ʹ-CAATGACCCCGCACGATTTC-3ʹ, reverse primer:5ʹ-CATGGAGGGCGGATTGGAA-3’; GAPDH forward primer:5ʹ-GAAGGTGAAGGTCGGAGTC-3ʹ, reverse primer:5ʹ-GAAGATGGTGATGGGATTTC-3’; U6 forward primer:5ʹ-CTCGCTTCGGCAGCACA-3ʹ, reverse primer:5ʹ-AACGCTTCACGAATTTGCGT-3ʹ.

### 2.5 Colony formation experiment

The NPC cells were trypsinized and then planted in 6-well plates (5 × 10^5^ cells/well). After the cells were cultured for 12 h, the cells were irradiated with 4 Gray (Gy) X-ray for 2 h and subsequently cultured at 37°C in 5% CO_2_ for 2 weeks. Subsequently, the medium was discarded, and PBS was used to wash the colonies 3 times. The colonies were dried, fixed for 30 minutes with 4% paraformaldehyde, and then stained for 10 minutes with a 0.5% crystal violet solution. The staining solution was gently washed away with tap water, and the plates were dried, and the number of colonies was observed by naked eyes.

### 2.6 CCK-8 experiment

The NPC cells were inoculated into 96-well plates (1000 cells per well), and the cells were treated with X-ray (0, 2, 4, 6, 8 Gy) for 2 h, and 10 μL of CCK-8 solution was supplemented into each well, and the cell culture was continued for another 1 h. Next, the absorbance of each well at 450 nm was measured by a microplate reader.

### 2.7 Flow cytometry

Apoptosis was measured using the Annexin V-FITC/PI double-labeled staining kit. 5–8 F-R and 6–10B-R cells treated with 4 Gy X-ray were harvested and then rinsed twice with PBS. The cells were suspended in binding buffer, then mixed with 5 μl of AnnexinV-FITC solution, and incubated for 15 min in the dark, and then 2.5 μL of PI solution was supplemented, and incubated for 5 min in the dark. Then the cells were rinsed by PBS, and detected by a flow cytometer (BD Biosciences, San Jose, CA, USA).

Cell cycle distribution was detected with PI solution. Briefly, 5–8 F-R and 6–10B-R cells were harvested and fixed by 70% ethanol for 12 h at 4°C. Subsequently, the cells were washed by PBS, and incubated with 50 μg/mL PI, 100 μg/mL RNase A and 0.2% Triton X-100 for 30 min at 4°C in the dark. Then the cells were cleaned by PBS, and detected by a flow cytometer (BD Biosciences, San Jose, CA, USA).

### 2.8 Dual-luciferase reporter gene assay

CDK1 3ʹUTR target sequence for miR-195-3p was inserted downstream of the firefly luciferase gene of the luciferase reporter vector, to construct wild-type CDK1 reporter (CDK1-WT) and mutant type CDK1 reporter (CDK1-MUT). The dual-luciferase reporter vectors were co-transfected into NPC cells with miR-195-3p mimics or NC mimics. 48 h later, the luciferase reporter gene experiment was executed according to the instructions of Dual-Luciferase Reporter Assay System, and firefly and Renilla fluorescence values were detected using a microplate reader, and Renilla fluorescence values were used as the internal reference.

### 2.9 Western blot

NPC cells were lysed in RIPA lysis buffer containing protease inhibitors, and the supernatant was collected after high-speed centrifugation. After protein extraction, protein concentrations were measured with the BCA Protein Assay Kit. Equal amounts of proteins (30 μg/group) were separated by SDS-PAGE and transferred to PVDF membranes. Subsequently, the proteins were blocked in 5% skim milk and maintained for 1 h. Next, the primary antibody anti-CDK1 (ab201008, 1:1000) was added to interact with the protein overnight at 4°C. Thereafter, the membranes were rinsed with Tris Buffered Saline Tween (TBST), and then the proteins were interacted with secondary antibody goat anti-rabbit IgG H&L (ab150077, 1:2000) for 1 h at room temperature. After washing the membrane 3 times, an electrochemiluminescence kit was added onto the membranes to show the bands, and GAPDH was the internal reference.

### 2.10 Gene set enrichment analysis (GSEA)

Enrichment analysis was performed with GSEA software (http://www.gsea-msigdb.org/gsea/index.jsp). NPC samples of TCGA database were grouped into high and low CDK1 expression groups based on the media value of CDK1 expression. 1000 gene set permutations were presented. According to the normalized enrichment score (NES), normalized p-value and false discovery rate (FDR), the potential pathways/biological processes which were associated with CDK1 expression, were predicted. The absolute value of NES ≥1.0, normalized p-value ≤ 0.05 and FDR ≤ 0.25 were used as the criteria to screen the gene sets.

### 2.11 Statistical analysis

SPSS 20.0 software (SPSS, Inc., Chicago, IL, USA) was exploited to analyze the data, and GraphPad Prism 9 was utilized to plot the figures. The student’s *t*-test was adopted to compare the data of two groups, and a one-way ANOVA with post-hoc test was evaluate to evaluate differences across three or more groups. *P* < 0.05 signified statistical significance.

## 3. Results

In the present work, the expression characteristics of miR-195-3p in raidoresistant NPC tissues and cell lines were investigated, and the regulatory effects of miR-195-3p on CDK1 are studied. The results of i*n vitro* experiments showed that the dysregulation of miR-195-3p/CDK1 axis contributes to the radioresistance of NPC.

### 3.1 MiR-195-3p expression in NPC tissues and cells and its relationship with clinicopathological characteristics

**Figure 1.**
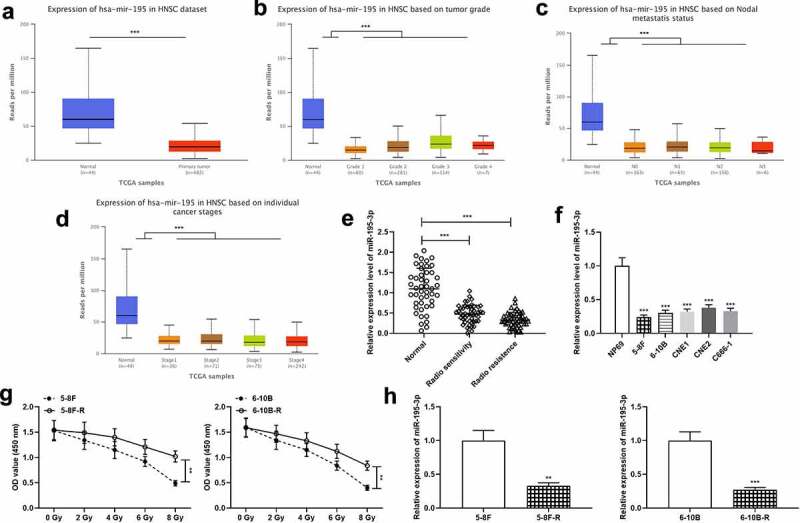
miR-195-3p is lowly expressed in NPC tissues and cells

UALCAN database (ualcan.path.uab.edu/home) exhibited that miR-195-3p was remarkably down-modulated in NPC tissue specimens, and was linked to increased tumor grade, lymph node metastasis and increased clinical stage of the NPC patients (Figure 1a-d). Next, miR-195-3p expression was examined in the normal tissues, radiosensitive NPC tissues and radioresistant NPC tissues, respectively by qRT-PCR. The data revealed that miR-195-3p expression was remarkably down-modulated in tumor tissues relative to the control group, and miR-195-3p expression was markedly down-modulated in the radioresistant group relative to the radiosensitive group (Figure 1e). Moreover, miR-195-3p expression was further measured in NPC cell lines, and the findings revealed that miR-195-3p expression was remarkably down-modulated in NPC cells (figure 1f). Furthermore, 5–8 F, 5–8 F-R, 6–10B and 6–10B-R cells were treated with different doses of radiation, and the results showed that the survival rate of 5–8 F-R and 6–10B-R cells was significantly higher compared to that of 5–8 F and 6–10B cells (Figure 1g). Additionally, qRT-PCR revealed that miR-195-3p expression was notably lower in 5–8 F-R and 6–10B-R cells compared with 5–8 F and 6–10B cells (Figure 1h).

A. UALCAN database was used to analyze the differences in miR-195-3p expression in NPC tissue samples and normal tissues.B-D. The relationship between miR-195-3p expression and tumor grade, lymph node metastasis and clinical stage of NPC patients was analyzed using UALCAN database.E. qRT-PCR was used to detect miR-195-3p expression in the normal tissues (n = 46), radiosensitive NPC tissues (n = 49) and radioresistant NPC tissues (n = 50).F. MiR-195-3p expression in human normal nasopharyngeal epithelial cell line NP69 and NPC cell lines (5–8 F, 6–10B, CNE1, CNE2, C666-1 cells) was detected by qRT-PCR.G. CCK-8 assay was used to detect the viability of 5–8 F-R cells and 6–10B-R cells under different doses of irradiation.H. MiR-195-3p expression in 5–8 F-R cells and 6–10B-R cells was detected by qRT-PCR, compared with the wild type cell lines.*p* < 0.05, ** p < 0.01, *** p < 0.001.

### 3.2 Up-regulation of miR-195-3p inhibits NPC cell growth, induces apoptosis and cell cycle arrest, and improves radiosensitivity

**Figure 2.**
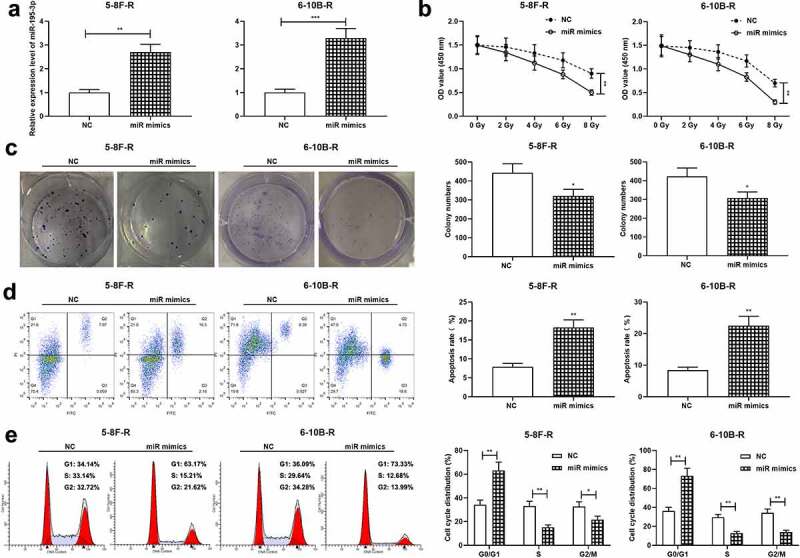
Effect of miR-195-3p on growth, apoptosis, cell cycle and radiosensitivity of NPC cells

Next, 5–8 F-R cells and 6–10B-R cells were transfected with miR-195-3p mimics and the control mimics, respectively, and qRT-PCR validated the successful transfection (Figure 2a). After treatment with different doses of radiation, cell viability was examined by the CCK-8 assay, and the data revealed that miR-195-3p overexpression significantly restrained cell viability (Figure 2b). Additionally, 4 Gy X-ray was utilized to irradiate the transfected cells, and colony formation experiment and flow cytometry showed that miR-195-3p overexpression restrained the viability, promoted apoptosis, and impeded cell cycle progression of NPC cells, relative to the control group (Figure 2b-e).

A. MiR-195-3p expression in 5–8 F-R and 6–10B-R cells transfected with NC mimics or miR-195-3p mimics was detected by qRT-PCR.B. After transfection, the cells were treated with different doses of radiation, and CCK-8 method was used to detect the viability of the cells in each group.C. After transfection, the effect of 4 Gy irradiation on the survival of the cells was examined using colony formation assay.D, E. After transfection, the effect of 4 Gy irradiation on the apoptosis and cell cycle of the cells was detected by flow cytometry.*p < 0.05, **p < 0.01, ***p < 0.001.

### 3.3 CDK1 is a direct downstream target of miR-195-3p

**Figure 3.**
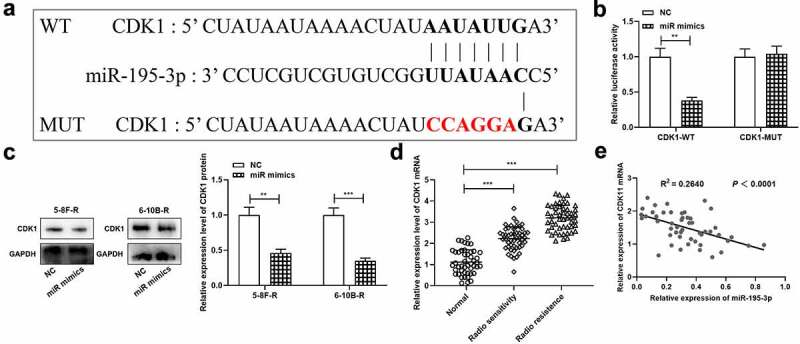
CDK1 is the target gene of miR-195-3p in NPC cell

To decipher the mechanism of miR-195-3p, CDK1 was projected as a downstream target of miR-195-3p by TargetScan database, and its binding sequence was shown (Figure 3a). Moreover, dual-luciferase reporter gene assay unveiled that up-regulation of miR-195-3p remarkably inhibited the luciferase activity in CDK1-WT group relative to the NC group, while in the CDK1-MUT group, there was no remarkably difference in luciferase activity. (Figure 3b). Western blot manifested that miR-195-3p overexpression down-modulated CDK1 expression in NPC cells (Figure 3c). Besides, the data of qRT-PCR showed that, miR-195-3p expression level was remarkably higher in the NPC tissues of the radioresistance group compared with that in the NPC tissues of the radiosensitive group (Figure 3d), and CDK1 mRNA expression level was negatively associated with miR-195-3p expression level in NPC tissues (Figure 3e).

A. The binding site between CDK1 mRNA and miR-195-3p.B. After co-transfection of dual-luciferase reporter plasmids and NC mimics or miR-195-3p mimics into 293 T cells, dual-luciferase reporter gene assay was used to detect the luciferase activity.C. After transfection of 5–8 F-R and 6–10B-R cells with NC mimics or miR-195-3p mimics, CDK1 protein expression in each group was detected by Western blot.D. CDK1 mRNA expression in normal tissues (n = 46), radiosensitive NPC tissues (n = 49) and radioresistant NPC tissues (n = 50) were detected by qRT-PCR.E. Correlation test was performed to analyze the correlation between CDK1 mRNA expression and miR-195-3p expression in NPC tissues of the radioresistant group.*p < 0.05, **p < 0.01, ***p < 0.001.

### 3.4 CDK1 overexpression reverses the effect of miR-195-3p overexpression on radiosensitivity of NPC cells

**Figure 4.**
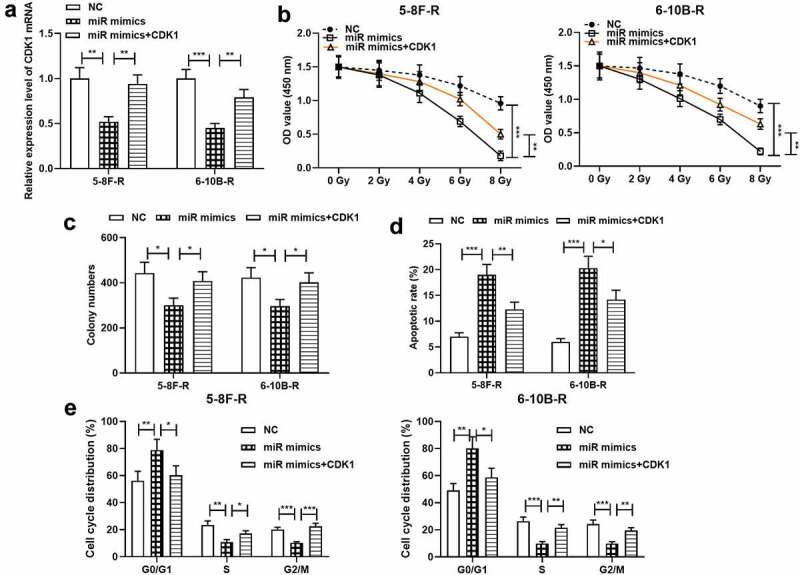
Effect of miR-195-3p and CDK1 on radiosensitivity of NPC cells

To further probe the effects of miR-195-3p and CDK1 on NPC cell growth, apoptosis, cell cycle and radiosensitivity, NC mimics, miR-195-3p mimics and miR-195-3p mimics + CDK1 overexpression plasmids were transfected into 5–8 F-R and 6–10B-R cells, respectively, and qRT-PCR validated the successful transfection (Figure 4a). Subsequently, CCK-8 experiment, colony formation experiment and flow cytometry showed that miR-195-3p overexpression remarkably inhibited cell growth, induced apoptosis and blocked the cell cycle progerssion relative to the control group, while the restoration of CDK1 expression partially reversed these effects (Figure 4c-e).

A. After transfection of NC mimics, miR-195-3p mimics and CDK1 overexpression plasmids into 5–8 F-R and 6–10B-R cells, respectively, CDK1 mRNA expression was detected by qRT-PCR.B. The effect of different doses of irradiation on the growth of the cells was examined by the CCK-8 method.C. The effect of 4 Gy irradiation on the survival of the cells was examined using colony formation assay.D, E. The effect of 4 Gy irradiation on the apoptosis of the cells was detected by flow cytometry.*p < 0.05, **p < 0.01, ***p < 0.001.

### 3.5 CDK1 activates pathways related with DNA replication and cell cycle

**Figure 5.**
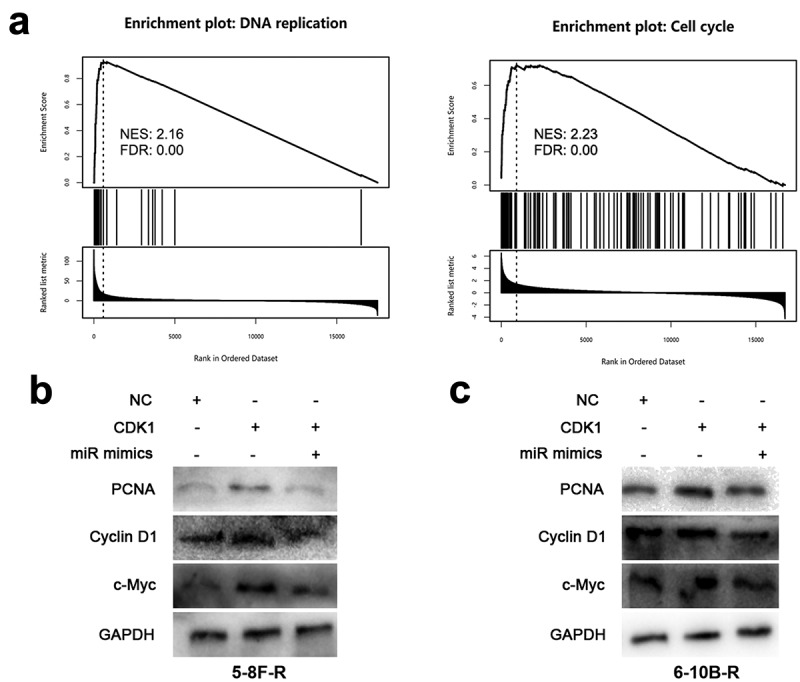
MiR-195-3p/CDK1 axis inhibits DNA replication and cell cycle related pathways

Next, GSEA was performed to understand the mechanism by which CDK1 affects the biological behaviors of NPC cells, and the data showed that CDK1 overexpression was associated with DNA replication and cell cycle related pathways (Figure 5a). To validate this, the effects of miR-195-3p and CDK1 on DNA replication and cell cycle-related proteins including PCNA, Cyclin D1 and c-Myc were examined by Western blot, and the results manifested that CDK1 overexpression resulted in augmented PCNA, Cyclin D1 and c-Myc expression, while miR-195-3p overexpression suppressed PCNA, Cyclin D1 and c-Myc expression (Figure 5b, c). These results implied that the miR-195-3p/CDK1 axis could disrupt DNA replication and cell cycle progression to regulate the radiosensitivity of NPC cells.

A. GSEA plot showed that CDK1 expression was associated with DNA replication and cell cycle-related pathways.B, C. After transfection of NC mimics, miR-195-3p mimics and CDK1 overexpression plasmids into 5–8 F-R and 6–10B-R cells, respectively, Western blot was adopted to detect PCNA, cyclin D1 and c-Myc expression.

## 4. Discussion

Radiotherapy is mainly used to inhibit the progression of tumors by inducing the injury of DNA of cancer cells, which leads to apoptosis and irreversible cell cycle arrest; cancer cells with resistance to radiotherapy will lead to cancer recurrence, metastasis and even the death of the patients [13,14]. Therefore, the sensitivity of NPC cells to radiation is one of the key factors affecting the prognosis of the patients [15,16]. It is crucial to find biomarkers for predicting the efficacy of radiotherapy, and the targets for improving the radiosensitivity of cancer cells.

MiRNA directs the silencing complex to degrade mRNA or block its translation by base-pairing with the 3ʹ UTR of the target gene’s mRNA [17]. A variety of biological behaviors of tumor cells are regulated by miRNAs, such as differentiation, growth, apoptosis, migration, invasion, drug resistance and radioresistance [6,18]. It is reported that miR-24-3p can modulate both 3ʹUTR and 5ʹUTR of Jab1/CSN5 to impede NPC cell growth and improve the radiosensitivity of NPC cells [19]. MiR-195 plays an inhibitory role in certain types of tumors such as gastric carcinoma and lung carcinoma [20,21]. MiR-195 has two mature sequences: miR-195-5p and miR-195-3p [22]. MiR-195-3p is transcribed from 17p13.1, and accumulating studies support that it is a modulator in cancer biology. For instance, miR-195-3p is overexpressed in renal cell cancer tissues and is associated with disease progression [23]. Downregulation of miR-195-3p expression in cervical cancer tissues is linked to adverse prognosis of the patients; miR-195-3p overexpression impedes cancer cell growth by targeting BCDIN3D [24]. In this work, miR-195-3p under-expression was validated to be correlated with adverse clinicopathological indicators of NPC patients. Intriguingly, in the tumor tissues with radioresistant characteristics, miR-195-3p expression was much lower, and in radioresistant NPC cell lines, miR-195-3p expression was also reduced. I*n vitro* experiments suggested that miR-195-3p overexpression restrained NPC cell growth, enhanced apoptosis, impeded cell cycle in G0/G1 phase, and enhanced radiosensitivity of NPC cells. The findings imply that miR-195-3p is tumor-suppressive in NPC progression, and its low expression hints radioresistance, and up-regulation of miR-195-3p increases the sensitivity of NPC cells to radiation.

CDK1 is an important member of the CDK family, consisting of catalytic kinase subunits and cell cycle protein chaperones [25]. CDK1 plays an important role in spindle morphogenesis and mitosis: the activated cyclin E-CDK1 complex promotes the cell cycle from G1 phase to S phase; after the cells entering the S phase, CDK1 promotes G2/M transition by forming a complex with cyclin A [26,27]. When CDK1 expression is abnormally activated, it leads to cell cycle deregulation, which in turn leads to abnormal cell growth, differentiation and apoptosis, and ultimately to tumorigenesis [28]. CDK1 expression is increased in most tumors, including NPC, and CDK1 inhibitors significantly inhibit tumor growth [29–31]. For example, synergistic effects of CDK1 inhibitors and MEK/ERK inhibitors can promote the apoptosis of colorectal cancer cells [31]. Dysregulated CDK1 expression in breast cancer is closely related with tumor development, and CDK1 overexpression facilitates the growth, migration and invasion of cancer cells [32]. The function of CDK1 in NPC has also been reported. Specifically, it is reported that tetrandrine significantly inhibits the growth of NPC cells and enhances radiosensitivity via regulating CDC25C/CDK1/cyclin B1 pathway [33]. According to another research, CDK1 is overexpressed in NPC and is closely associated with adverse clinicopathological parameters of the patients; miR-96-5p induces apoptosis of NPC cells by targeting CDK1, and improves the radiosensitivity of NPC cells [29]. In this work, CDK1 was demonstrated to be a downstream target of miR-195-3p, and it was also revealed that miR-195-3p/CDK1 axis regulated the radiosensitivity of NPC cells, and PCNA, Cyclin D1 and c-Myc expression, which partly explains the mechanism of miR-195-3p in the raidoresistance of NPC cells.

## 5. Conclusion

In summary, this work report that miR-195-3p overexpression enhances the radiosensitivity of NPC cells and inhibits tumor growth by targeting CDK1. This study further elucidates the molecular mechanism of radioresistance of NPC cells, which provides useful information for reversing the radioresistance of NPC and improving radiotherapy efficacy.

## Data Availability

The data used to support the findings of this study are available from the corresponding author upon request.

## Research highlights

1. MiR-195-3p expression level is down-regulated in radiation-resistant NPC tissues and NPC cells with radioresistance.
2. CKD6 is a direct target of miR-195-3p in NPC cells.
3. MiR-195-3p/CDK6 axis modulates the radiosensitivity of NPC cells.
